# Breastfeeding Needs in Adolescent Mothers: A systematic review

**DOI:** 10.18295/squmj.12.2023.090

**Published:** 2024-08-29

**Authors:** Atefeh Yas, Fatemeh Z. Karimi, Talat Khadivzadeh

**Affiliations:** 1 School of Nursing and Midwifery, Mashhad University of Medical Science, Mashhad, Iran; 2 Nursing and Midwifery Care Research Center, Mashhad University of Medical Sciences, Mashhad, Iran; 3 Department of Midwifery, Mashhad University of Medical Science, Mashhad, Iran

**Keywords:** Breastfeeding, Adolescent Mothers, Qualitative Study, Systematic Review

## Abstract

Adolescent mothers face numerous challenges while breastfeeding. This study aimed to assess the breastfeeding needs of adolescent mothers. For this systematic review, Web of Science, PubMed, Scopus, Cochrane Library, SID and Magiran databases were searched. The initial search yielded 2,290 studies, of which 41 were included in this review. Adolescent mothers’ breastfeeding requirements were grouped into 8 categories: (1) social support from healthcare providers, partners and families; (2) school support for breastfeeding mothers; (3) breastfeeding counselling based on cultural sensitivities; (4) educational assistance from health providers for adolescent mothers' families; (5) changing harmful cultural values and judgments about adolescent mothers' breastfeeding; (6) additional home or outpatient visits in the days following hospital discharge; (7) peer support and counselling; and (8) economic needs. To promote breastfeeding, policymakers and healthcare providers should devise specifically tailored programmes and interventions to cater to the specific requirements of adolescent mothers.

Breastfeeding is the most effective method of ensuring that infants receive the necessary nutrients for optimal growth. Moreover, breastfeeding provides numerous benefits for infants, including promoting their overall health and reducing their risk of developing several chronic diseases. Breastfed infants have a lower incidence of acute otitis, gastroenteritis, severe respiratory tract infections, atopic dermatitis, asthma, obesity, types 1 and 2 diabetes, childhood leukaemia, sudden infant death syndrome and necrotising enterocolitis.^1^^,^^2^ The World Health Organization recommends exclusive breastfeeding for the first 6 months of life, followed by complementary foods and continued breastfeeding until the child reaches 2 years of age.^3^^,^^4^

Breastfeeding mothers are affected by various factors, including the mother's age.^5^ According to various studies, the rate of exclusive breastfeeding among adolescent mothers is lower than that among adult mothers, with a prevalence of 22% in Canadian adolescent mothers compared to 51% in Canadian adult mothers.^6^ In Thailand and Bangladesh, 19% and 17.3% of adolescent mothers exclusively breastfeed their infants until 6 months of age.^7^^,^^8^ In the United States, 19.3% of mothers aged 20 years exclusively breastfeed their infants for the first 3 months of life.^9^^,^^10^

Adolescent mothers face numerous changes and challenges while pregnant and breastfeeding and must adjust to adult social roles, the physical changes associated with puberty and the struggles of raising an infant. Most of these mothers are in poor social and economic circumstances and face numerous physical, mental, social and spiritual challenges, such as depression, anxiety, low self-efficacy, lack of self-confidence and multiple responsibilities; caring for their children and breastfeeding become a struggle for adolescent mothers as they encounter these obstacles.^11^^,^^12^ An adolescent mother also experiences several adverse health consequences.^13^ Evidence suggests that adolescents have a higher rate of obstetric complications as well as low-birth-weight infants, exposing their infants to disease and mortality risk.^14^^,^^15^ Breast milk is the best nutrition for infants because it protects them from numerous diseases and promotes their sensory and cognitive development.^16^

The WHO also referred to adolescent mothers' breastfeeding as ‘feeding in exceptionally challenging circumstances’, classifying them as high-risk mothers.^17^ Therefore, it is essential to understand these mothers’ breastfeeding requirements to promote breastfeeding among them and design health policies and interventions specific to their needs. To the best of the authors’ knowledge, no systematic review has examined all facets of the breastfeeding requirements of adolescent mothers. Thus, the current systematic review aimed to determine the breastfeeding needs of adolescent mothers across qualitative studies conducted in low-, middle- and high-income countries.

## Methods

This review followed the Preferred Reporting Items for Systematic Reviews and Meta-Analyses (PRISMA) guidelines. The 2009 PRISMA statement was designed to assist systematic reviewers in reporting their review's motivation, the authors' methods and their findings in a transparent manner. An update to the guideline was necessary due to developments in systematic review terminology and methodology over the past 10 years. Therefore, the PRISMA 2020 statement was developed to replace the 2009 statement, and it entails a new reporting guideline that reflects improvements in the methods of identifying, evaluating and synthesising studies.^18^

### DATA SOURCES AND SEARCH STRATEGY

Two researchers independently reviewed relevant articles with a qualitative approach in both Persian and English that were published until December 2022. Web of Science, PubMed, Scopus and the Cochrane Library databases were searched for English language articles, while the SID and Magiran databases were searched for articles written in Persian. The key phrases were a combination of the following terms or their MeSH counterparts with Boolean operators (‘AND’ or ‘OR’): ‘breastfeeding’, ‘lactation’, ‘infant feeding’, ‘teenage mothers’, ‘adolescent mothers’, ‘young mothers’, ‘needs’ and ‘qualitative study’. Additionally, the reference sections of the relevant articles were manually examined to discover overlooked interventions.

### ELIGIBILITY CRITERIA

Qualitative and mixed-method studies that addressed the breastfeeding requirements of adolescent mothers with full-term, healthy new-borns were included in this review. Case reports, review studies, letters to the editor, systematic reviews, studies with unrelated data, quantitative studies and articles without full text were excluded.

Ethnography, phenomenology, the grounded theory, qualitative descriptive research and qualitative content analysis are a few of the qualitative study methods frequently used in the medical and nursing sciences.^19^

Ethnography is the literal writing of culture and refers to a direct description of a community, group or culture. Data are gathered while working in the field, through participant observation, field note-taking, interviews with key informants and document collection. Phenomenology is an approach to philosophy and a well-known qualitative research methodology. It aims to describe specific phenomena of daily experience as lived experience in order to comprehend their fundamental structure (i.e. essence). The grounded theory is a systematic approach to studying social processes. It originates from sociology, specifically from symbolic interactionism and has the development or modification of theory as its goal. In qualitative descriptions (QD), the what, who and where of the experiences are the main focus. The core or foundation of QD differs from that of other varieties of qualitative research in that its primary focus is descriptive rather than interpretive. Qualitative content analysis is a research technique used to subjectively interpret the content of text data through the systematic classification process of coding and identifying categories, themes or patterns.^19^

### STUDY SELECTION AND DATA COLLECTION PROCESS

Studies found in the electronic databases were sent to Endnote X8 (Clarivate Plc, Philadelphia, USA) for duplicate removal, screening and assessment. The titles, abstracts and keywords of the articles, as well as their eligibility criteria and selection of the eligible studies were independently evaluated done by 2 authors (AY and FZK). Next, the complete text of the selected publications was independently examined by 2 authors (AY and FZK), and any disagreements were resolved by discussion with a third author (TKH).

Extracted data included the author's name, year of publication, country where the study was conducted, type of qualitative approach, article title and the type of breastfeeding reported in the article [Table 1].

### EVALUATION OF THE QUALITY OF STUDIES

The Critical Appraisal Skills Program (CASP) checklist for qualitative studies was used to assess the quality of the selected studies.^20^ This tool evaluates all articles using qualitative methodology, and it is used for quality assessment in health and social care-related qualitative evidence synthesis.^21^ The CASP checklist used in this review consisted of items, aims, methodology, data collection process, reflexivity, ethical considerations, analytical process, findings, and the value placed on the research.

The quality of the studies was independently assessed by 2 authors (AY and FZK). As the CASP checklist does not have a ranking system, the authors decided to assign a score of 0 for ‘no’, 1 for ‘can't say’ and 2 for ‘yes’ to facilitate the grading of the studies; the score ranged from 0–20. In the current review, all retrieved articles scored 15 or higher. The results of all the included studies were valid, and the design of all studies was appropriate for addressing the aims of the research.

## Results

**Figure 1:**
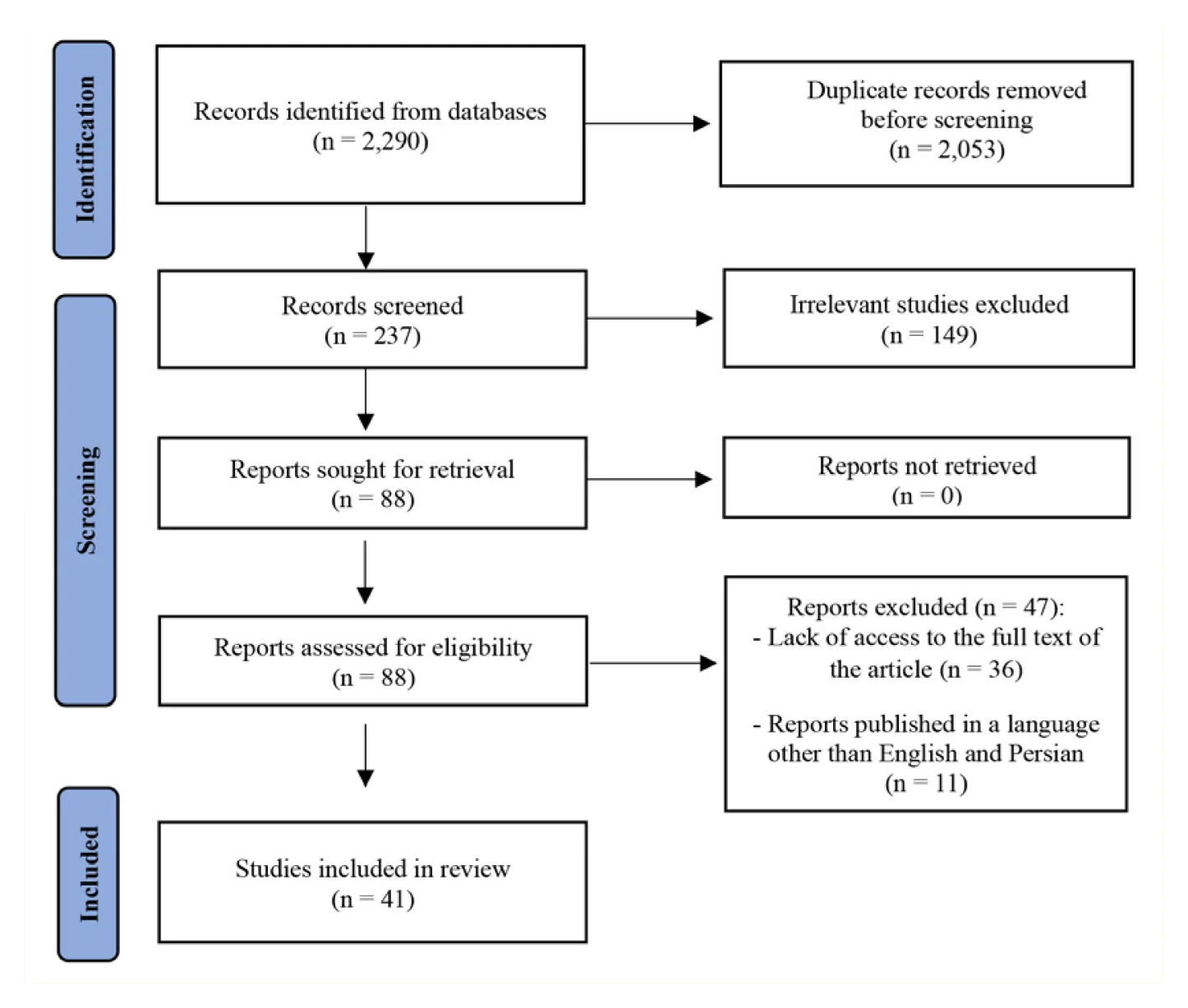
Flow diagram showing how the studies were identified in the current systematic review.

A total of 2,290 studies were retrieved from the various databases searched, of which 2,053 were excluded due to duplication. Abstracts and titles were examined for the 237 studies and 149 were excluded for being unrelated. The remaining 88 studies were deemed eligible and their full texts were reviewed; finally, 41 studies were included in this systematic review [Figure 1]. The selected articles were published between the years 2000 and 2022; 10 articles were published in the USA, 8 in the UK, 5 in Canada, 5 in Brazil, 3 in Indonesia, 2 in Thailand, 2 in Ghana, 2 in South Africa, 1 in Iran, 1 in Iceland, 1 in Belgium and 1 in Australia [Table 1].^11^^,^^22^–^61^ The age range of breastfeeding mothers in all the studies was 12–18 years.

After reviewing the study findings, the different breastfeeding needs of adolescent mothers were classified into the following eight categories: (1) social support from healthcare providers, partners and families; (2) school support for breastfeeding mothers; (3) breastfeeding counselling based on cultural sensitivities; (4) educational assistance from health providers for adolescent mothers' families; (5) changing harmful cultural values and judgments about adolescent mothers' breastfeeding; (6) additional home or outpatient visits in the days following hospital discharge; (7) peer support and counselling; and (8) economic needs.

### SOCIAL SUPPORT FROM HEALTHCARE PROVIDERS, PARTNERS AND FAMILIES

To overcome the physical and psychological challenges of breastfeeding, mothers require assistance from their families, sexual partners and healthcare providers.^11^^,^^40^^,^^45^^,^^52^ Access to health centres and guidance from healthcare professionals is imperative for these mothers. Before and after delivery, they require support and guidance to ensure their well-being and address any challenges they may face during breastfeeding.^23^^,^^37^^,^^39^^,^^42^^,^^46^^,^^47^^,^^61^ Moreover, the support of family members, particularly that of their own mothers, is crucial for these new mothers during the postpartum period and throughout breastfeeding.^34^^,^^49^^,^^55^^,^^61^ The supportive needs reported by adolescent mothers include emotional support, self-esteem enhancement, practical assistance, informative resources and dependable support networks.^24^^,^^35^^,^^43^

### EMOTIONAL SUPPORT

Emotional support entails expressing empathy, providing consolation and attentively listening. Adolescent mothers require healthcare providers and families to reassure them about their breastfeeding experiences, empathise with them and listen to their problems and needs.

### SELF-ESTEEM ENHANCEMENT

Breastfeeding and appropriately tending to their child requires adolescent mothers to cultivate self-worth, believe in themselves and refrain from criticising their abilities as mothers.

### INSTRUMENTAL/PRACTICAL SUPPORT

Receiving instrumental assistance during breastfeeding involves obtaining tangible and practical aid. Adolescent mothers require monitoring from healthcare professionals, who will address their breastfeeding concerns through effective practical guidance.

### EDUCATIONAL AND INFORMATIONAL SUPPORT

Adolescent mothers need to assess their breastfeeding knowledge and receive accurate information regarding the benefits of breast milk for infants, techniques for breastfeeding and solutions for resolving any breastfeeding difficulties.^22^^,^^23^^,^^40^^,^^44^^,^^46^^,^^48^^,^^50^^,^^52^^,^^58^^,^^60^^,^^61^

### NETWORK SUPPORT

To overcome the responsibilities of caring for the infant and breastfeeding, adolescent mothers require network support from family, friends, sexual partners and healthcare providers.^26^^,^^36^^,^^38^^,^^53^

### SCHOOL SUPPORT FOR BREASTFEEDING MOTHERS

Adolescent mothers should be supported in their pursuit of education following childbirth. Additionally, a facility should be available for them to store their expressed milk while attending school. These mothers require constant guidance regarding the timeline for returning to school after childbirth and the availability of appropriate childcare facilities.^22^^,^^25^^,^^30^^,^^32^^,^^41^^,^^44^^,^^47^^,^^53^^,^^56^^,^^57^

### ADDITIONAL HOME OR OUTPATIENT VISITS IN THE DAYS FOLLOWING HOSPITAL DISCHARGE

This implies that adolescent mothers will have an additional visit either at their residence or at a medical facility, shortly after their hospital discharge. This additional visit will provide them with practical assistance and facilitate their ability to nurse their infant.^22^

### EDUCATIONAL ASSISTANCE FROM HEALTH PROVIDERS FOR ADOLESCENT MOTHERS' FAMILIES

Due to their lack of independence, adolescent mothers often have limited control in determining their baby's nutritional needs. Within the household, grandmothers often make the decisions, and adolescent mothers have complete confidence in their judgment, adhering to their advice. Adolescent mothers believe their own mothers have more experience caring for and nursing babies, so they defer to them for advice and support. To ensure proper nourishment for the baby, grandmothers should acquire knowledge on breastfeeding and receive education on the subject.^11^^,^^56^

### CHANGING HARMFUL CULTURAL VALUES AND JUDGMENTS ABOUT ADOLESCENT MOTHERS' BREASTFEEDING

Adolescent mothers' experiences with local cultures regarding breastfeeding and infant feeding methods and the social judgments they face in society are referred to as ‘cultural values’ and ‘judgments’. According to some studies, adolescent mothers' breastfeeding methods are influenced by local cultures and the judgment of other members of society. In many of the included studies, adolescent mothers mentioned that the cultural practices in their community and the judgments of others affect their breastfeeding methods.^25^–^28^^,^^34^^,^^41^ Adolescent mothers often feel obligated to breastfeed (regardless of their personal preferences) due to cultural values, attitudes and judgment by members of society as well as health professionals. According to some adolescent mothers, they frequently receive unwanted remarks and societal stigma from strangers when breastfeeding their babies in public.^47^^,^^52^^,^^61^

### PEER SUPPORT AND COUNSELLING

Peer assistance and counselling are necessary for adolescent mothers postpartum and during breastfeeding. They have a greater comfort level when conversing with friends rather than with professionals.^25^^,^^43^

### RECEIVING BREASTFEEDING COUNSELLING BASED ON CULTURAL SENSITIVITIES

To ensure appropriate support, it is crucial for healthcare providers to establish strong communication channels with adolescent mothers and have a deep comprehension of the obstacles they face while breastfeeding. When offering breastfeeding training and counselling, healthcare providers must acknowledge and respect each mother’s cultural background and sensitivities to ensure that the support is fitting and considerate.^32^

### ECONOMIC NEEDS

Due to insufficient parental funds, teenage mothers often require financial assistance to afford essential items such as medication, groceries, personal items and transportation. Thus, it becomes necessary for these young mothers to seek employment to cover their expenses and provide for their children.^44^

## Discussion

This review aimed to determine the type of assistance that adolescent mothers require regarding breastfeeding. Information was gathered from 41 studies, and the current review's findings revealed 8 different categories of breastfeeding needs among adolescent mothers, including the need for social support from family, sexual partners, friends and medical professionals during breastfeeding. The difficulties of puberty, the ensuing psychological and physical changes and adolescent mothers' ignorance of infant care and breastfeeding all contribute to their increased dependence on others.^54^^,^^62^^,^^63^ For adolescent mothers in Indonesia and the USA, support from family and friends, particularly parents, is essential for initiating and continuing breastfeeding. Successful breastfeeding requires the support of family members, the provision of accurate information, motivation and the establishment of friendships for these mothers.^31^^,^^64^^,^^65^

Adolescent mothers in Thailand who receive assistance from their families are more inclined to initiate and maintain breastfeeding for some time.^66^ In both the USA and England, adequate maternal grandmother support fosters effective parenting and adolescent adaptation to the maternal role, which simultaneously influences the benefits of breastfeeding.^30^^,^^38^^,^^59^ Support from a sexual partner is additionally recognised as a significant facilitator of breastfeeding continuation in adolescent mothers. According to research from Ghana, Hawaii and Indonesia, sexual partners' verbal encouragement and participation in breastfeeding improves adolescent mothers' ability, confidence and self-esteem to breastfeed.^27^^,^^55^^,^^67^^,^^68^

Adolescent mothers require continuous support from healthcare professionals to overcome barriers to breastfeeding.^24^^,^^35^ Formal support in the early postpartum period and providing a supportive setting in which to learn to breastfeed are beneficial for increasing adolescent mothers’ knowledge, skills, self-confidence and sense of empowerment while breastfeeding.^55^^,^^69^^,^^70^ Ingram *et al*. discovered that young Somali and Afro-Caribbean women had minimal knowledge of the benefits of breast milk.^71^ The absence of practical breastfeeding knowledge can lead to dissatisfaction, diminished self-assurance and an early initiation of milk powder.^37^^,^^44^ According to a survey of 53 adolescent mothers in Virginia, USA, most mothers do not receive adequate information about the benefits of breastfeeding after giving birth.^72^ According a 2004 study by Wambach and Koehn in the USA, adolescent mothers should be informed about common breastfeeding misconceptions, including the idea that breastfeeding is related to breast size and that breastfeeding causes a mother's breasts to sag.^73^

Another need expressed by breastfeeding adolescent mothers is the provision of educational support to their households by health professionals. Because adolescent mothers lack independence, they may live with their family members, and thus, their breastfeeding practice is impacted by their own parents and other family members.^25^^,^^63^^,^^74^ Despite receiving adequate breastfeeding guidance from healthcare professionals, adolescent mothers in South Africa and Thailand were unable to breastfeed because grandmothers insisted on their opinions on how to feed the infant.^11^^,^^56^ For instance, according to 67.3% of Brazilian grandmothers, infants younger than 6 months of age should be given food, even though doing so increases their risk for diseases and symptoms such as diarrhoea, which can leave them weak, malnourished and critically ill.^75^ Adolescent mothers in Thailand usually abandon exclusive breastfeeding when faced with family conflicts related to infant feeding.^76^

Shifting cultural norms and judgments is one of the challenges adolescent mothers face during breastfeeding. Examples of behaviours that prevent breastfeeding initiation include giving the infant special foods or liquids and delaying early feeding. Studies in India and Kenya showed that people believe that infants should not be given colostrum or water during the first week of life to prevent jaundice.^77^^,^^78^

Further research conducted in Thailand and Brazil revealed that the social stigma associated with being an adolescent mother and the judgments made by others about her ability to breastfeed make breastfeeding challenging and complicated, particularly in public settings.^47^^,^^52^ The results of a 2010 systematic review by MacGregor and Hughes revealed that adolescent mothers frequently struggle with breastfeeding due to concerns about judgment by others and potential embarrassment, resulting in decreased self-assurance.^79^ Utilising mass communication tools to educate the public and alter societal attitudes towards adolescent mothers can alleviate the stigma that exists in society.

Peer support and counselling was another need expressed by adolescent mothers regarding breastfeeding. Adolescent mothers who have formal or informal relationships with their peers are more likely to succeed in exclusively breastfeeding their infants. Even supportive friends who may lack knowledge about breastfeeding play a crucial role in boosting the confidence of adolescent mothers and sustaining commitment to breastfeeding.^25^^,^^80^

It is often crucial to provide adolescent mothers with tailored support and counselling on breastfeeding based on their cultural and individual backgrounds. Breastfeeding support is essential as it allows adolescent mothers to express their fears and worries regarding their infant's feeding. Gaining insight into the individual requirements of adolescent mothers, offering them emotional support and enhancing their self-esteem is imperative. Encouraging them to breastfeed presents a valuable opportunity to foster a strong connection between mother and baby.^32^ Healthcare professionals conducting breastfeeding counselling sessions should display empathy and respect towards the individual requirements of each adolescent mother while also acknowledging and valuing their cultural beliefs.^81^^,^^82^

While breastfeeding, adolescent mothers require assistance from their educational institution. A collaboration between the education and health departments is needed to assist adolescent mothers in continuing their education while also breastfeeding. Their responsibility is to ensure that these mothers breastfeed their infants exclusively and receive the necessary support to return to school.^56^^,^^83^ In studies in the USA, Canada and South Africa, the lack of school support for adolescent mothers during breastfeeding led to the discontinuation of exclusive breastfeeding.^44^^,^^57^^,^^84^ To facilitate the return of adolescent mothers to school after giving birth, a kindergarten may be opened which is situated close to a school to care for the children; the cost of child care should also be waived. Adolescent mothers in this scenario will have the option of leaving their children at the kindergarten while they attend school. Teachers can also tutor adolescent mothers through online platforms or in-person interactions.

Adolescent mothers require financial support during breastfeeding. These mothers often experience poverty either because they lack the academic qualifications for specific job positions, financial support from their impoverished parents and funds to initiate a small business or because they struggle to secure employment due to pregnancy or having a child, which poses significant obstacles to their employment prospects.^44^ Training in skills such as farming and handicrafts can thus be an possibility to improve the economic status of these mothers.^85^ It is essential to mention that if an adolescent mother has the financial means, her access to food will improve.^86^ Furthermore, if an adolescent mother has financial means, she would have more time to fulfil her motherly responsibilities.

This review’s strength is its focus on various studies that have contributed to the current knowledge on adolescent mothers' breastfeeding needs. A limitation of this study is the inapplicability of its findings to poorer populations since most of the included studies were conducted in developed and high-income countries.

### RECOMMENDATION

Policymakers in the health system should pay attention to adolescent mothers' breastfeeding needs and design the necessary programmes and interventions to meet those needs. Ministries of education should enact the necessary policies and laws to encourage adolescent mothers to return to school. It is recommended that non-governmental organisations provide financial assistance to adolescent mothers and that future studies design and implement the necessary interventions based on the current research findings to address the breastfeeding needs of adolescent mothers.

## Conclusion

According to the current review's findings, teenage mothers have various requirements to successfully breastfeed. Families, partners, friends and healthcare providers play a vital role in providing the necessary support for successful breastfeeding. Furthermore, the cultural values and social stigma associated with being an adolescent mother must be addressed; therefore, supportive efforts should be made to support breastfeeding in public, reduce the stigma associated with being an adolescent parent and create a culture in which breastfeeding is accepted as a norm for all mothers. These mothers require assistance from their school to return after giving birth and exclusively breastfeed their babies. In addition, healthcare providers should improve adolescent mothers' breastfeeding training and knowledge to help these mothers breastfeed more effectively.

## Figures and Tables

**Table 1:** Characteristics of the articles included in the current review (N = 41)^11^^,^^22^–^61^

Author and year of publication	Study design	Country	Study title	Type of need	CASP score
Hannon *et al*.^28^ (2000)	Qualitative descriptive study	USA	African-American and Latina adolescent mothers’ infant feeding decisions and breastfeeding practices: A qualitative study	- Changing cultural values and judgments - Educational and skills needs about breastfeeding	16
Dykes *et al*.^35^ (2003)	Qualitative exploratory study	UK	Adolescent mothers and breastfeeding: experiences and support needs—An exploratory study	- Social support from health providers, partners and grand mothers	17
Nelson and Sethi^42^ (2005)	Grounded theory	Canada	The breastfeeding experiences of Canadian teenage mothers	- Social support from health providers	18
Hall Moran *et al*.^33^ (2006)	Qualitative descriptive study	UK	Breastfeeding support for adolescent mothers: Similarities and differences in the approach of midwives and qualified breastfeeding supporters	- Social support	18
Arthur *et al*.^36^ (2007)	Phenomenology	England	Teenage mothers’ experiences of maternity services: A qualitative study	- Social support from health providers and family	17
Morrison *et al*.^27^ (2008)	Qualitative study	USA	Determinants of infant-feeding choice among young women in Hilo, Hawaii	- Social support from health providers, partners and grandmothers - Changing cultural values and judgments	16
Wambach and Cohen^29^ (2009)	Qualitative descriptive study	USA	Breastfeeding experiences of urban adolescent mothers	- Educational and skills needs about breastfeeding - School support - Social support	16
Nelson^59^ (2009)	Qualitative exploratory study	Iceland	Adolescent attitudes, beliefs and concerns regarding breastfeeding	- Educational and skills needs about breastfeeding - Social support from health providers	16
Aujoulat *et al*.^60^ (2010)	Qualitative exploratory study	Belgium	Adolescent mothers’ perspectives regarding their own psychosocial and health needs: A qualitative exploratory study in Belgium	- Educational and skills needs about breastfeeding	18
Tucker *et al*.^22^ (2011)	Mixed Methods study	USA	Infant feeding experiences among teen mothers in North Carolina: Findings from a mixed method study	- Extra home visit - School support form breastfeeding mothers	19
Hansen and Wambach^30^ (2011)	Qualitative descriptive study	USA	Exploring barriers to exclusive breastfeeding among adolescent Latina women	- School support - Family support	16
Smith *et al*.^23^ (2012)	Qualitative prospective study	USA	Early breastfeeding experiences of adolescent mothers: a qualitative prospective study	- Educational and skills needs about breastfeeding - Social support from health providers and partner	18
Nesbitt *et al*.^40^ (2012)	Qualitative descriptive study	Canada	Canadian adolescent mothers’ perceptions of influences on breastfeeding decisions: a qualitative descriptive study	- Social support - Educational and skills needs about breastfeeding	18
Woods *et al*.^26^ (2013)	Qualitative study	USA	Describing Adolescent Breastfeeding Environments Through Focus Groups in an Urban Community	- Changing cultural values and judgments - Social support from health providers	15
Condon *et al*.^34^ (2013)	Qualitative descriptive study	UK	But is it a normal thing? Teenage mothers’ experiences of breastfeeding promotion and support	- Educational and skills needs about breastfeeding - Changing cultural values and judgments about breastfeeding of adolescent mothers	15
Pentecost and Grassley^24^ (2014)	Qualitative content analysis	USA	Adolescents’ Needs for Nurses’ Support When Initiating Breastfeeding	- Social support from health providers	18
Bettison^37^ (2014)	Phenomenology	England	Health visitors’ perceptions of encouraging and supporting teenage mothers to breastfeed	- Educational and skills needs about breastfeeding - Social support from health providers	17
Hunter^38^ (2014)	Qualitative study	England	Supporting teenage mothers to initiate breastfeeding and developing a support intervention to increase breastfeeding rates in a vulnerable group – the importance of place	- Social support from health providers	16
Monteiro *et al*.^45^ (2014)	Mixed-method study	Brazil	Breast feeding among Brazilian adolescents: Practice and needs	- Social support from health providers, partner and family	17
de Azevedo Mazza *et al*.^49^ (2014)	Qualitative exploratory study	Brazil	Influence of social support networks for adolescent breastfeeding Mothers in the process of breastfeeding	- Social support from health providers, partner and family	18
Hunter *et al*.^39^ (2015)	Qualitative study	England	Disempowered, passive and isolated: how teenage mothers’ postnatal inpatient experiences in the UK impact on the initiation and continuation of breastfeeding	- Social support from health providers	15
Hackett *et al*.^41^ (2015)	Qualitative descriptive study	Canada	A qualitative study exploring perceived barriers to infant feeding and caregiving among adolescent girls and young women in rural Bangladesh	- Educational and skills needs about breastfeeding - School support	18
Oliveira *et al*.^47^ (2016)	Qualitative descriptive study	Brazil	Breastfeeding exclusive breastfeeding: interruption of causes in mothers of teens perception	- Educational and skills needs about breastfeeding - Support and flexibility at work and school	16
Souza *et al*.^48^ (2016)	Qualitative stud	Brazil	Breastfeeding: factors affecting the early weaning between adolescent mothers	- Educational and skills needs about breastfeeding	17
Rossman *et al*.^31^ (2017)	Qualitative study	USA	Human Milk Provision Experiences, Goals, and Outcomes for Teen Mothers with Low-Birth-Weight	- Social support from health providers and grandmothers - Educational and skills needs about breastfeeding	18
Edwards *et al*.^43^ (2017)	Qualitative content analysis	Canada	Factors influencing the breastfeeding Practices of young mothers living in a maternity shelter: A qualitative study	Social and emotional support from health providers and peers	18
de Bairros Tamara *et al*.^46^ (2017)	Qualitative descriptive study	Brazil	Support received by adolescent mothers in the maternal breastfeeding process	- Educational and skills needs about breastfeeding - Social support from health providers, partner and family	16
Mangeli *et al*.^58^ (2017)	Qualitative content analysis	Iran	Exploring the challenges of adolescent mothers from their life experiences in the transition to motherhood	- Educational and skills needs about breastfeeding - Social support	18
Nabugoomu *et al*.^44^ (2018)	Qualitative descriptive study	Canada	Needs and barriers of teen mothers in rural eastern Uganda: stakeholders’ perceptions regarding maternal/child nutrition and health	- School support - Educational and skills needs about breastfeeding - Economic needs	17
Nuampa *et al*.^53^ (2018)	Qualitative descriptive Study	Thailand	Breastfeeding Experiences among Thai Adolescent Mothers: A Descriptive Qualitative Study	- Social support from family and health providers - School support - Changing cultural values and judgments	18
Jama *et al*.^56^ (2018)	Qualitative longitudinal design	South Africa	Autonomy and infant feeding decision making among teenage mothers in a rural and urban setting in KwaZulu-Natal, South Africa	- Support from health providers for family of adolescent mothers - School support form breastfeeding mothers	18
Chopel *et al*.^25^ (2019)	Qualitative prospective study	USA	Multilevel Factors Influencing Young Mothers’ Breastfeeding: A Qualitative CBPR Study	- Peer support and peer counselling - School support - Changing cultural values and judgments	19
Erfina *et al*.^51^ (2019)	Phenomenology	Indonesia	Exploring Indonesian adolescent women’s healthcare needs as they transition to motherhood: A qualitative study	- Educational and skills needs about breastfeeding - Social support from health providers	19
Thongmixay *et al*.^57^ (2023)	Qualitative study	South Africa	Isolation: The experience of adolescent motherhood	- School support form breastfeeding mothers	16
Jamie *et al*.^32^ (2020)	Qualitative study	UK	Healthcare practitioner relationships, cultural health capital and breastfeeding support for adolescent mothers	- Receiving breastfeeding counselling based on the cultural sensitivities - School support for breastfeeding mothers	18
Nuampa *et al*.^11^ (2020)	Qualitative descriptive study	Thailand	Breastfeeding challenges among Thai adolescent mothers: hidden breastfeeding discontinuation experiences	- Support from health providers for family of adolescent mothers - Social support from family and health providers	17
Acheampong *et al*.^54^ (2020)	Qualitative exploratory study	Ghana	Perceived enablers of exclusive breastfeeding by teenage mothers in Ghana	- Social supports from health providers and family - Educational and skills needs about breastfeeding	18
Wijaya *et al*.^50^ (2021)	Phenomenology	Indonesia	Qualitative Study of Breastfeeding Practice Experiences of Teenager Mothers	- Social support from health providers, partner and family - Educational and skills needs about breastfeeding	17
Astuti *et al*.^52^ (2021)	Qualitative exploratory study	Indonesia	A Qualitative Study on the Breastfeeding Experiences of Young Mothers	- Educational and skill needs about breastfeeding - Social support from family, partner and peers - Changing destructive cultural values	18
Twintoh *et al*.^55^ (2021)	Phenomenology	Ghana	Childcare practices among teenage mothers in Ghana: a qualitative study using the ecological systems theory	- Social support from health providers, partner and family	18
Buckland *et al*.^61^ (2022)	Qualitative study	Australia	Experiences of young Australian mothers with infant feeding	- Social support from health providers and peer - Educational and skills needs about breastfeeding - Changing cultural values and judgments	19

CASP = Critical Appraisal Skills Program.
